# Prolonged and Recurrent De Quervain’s Thyroiditis After Thyroid Fine Needle Aspiration

**DOI:** 10.7759/cureus.87767

**Published:** 2025-07-12

**Authors:** Kyrstin Lane, Brendan Pelikh, Jianyu Rao, Dianne S Cheung

**Affiliations:** 1 Endocrinology, Diabetes and Metabolism, David Geffen School of Medicine at University of California, Los Angeles (UCLA), Los Angeles, USA; 2 Endocrinology, University of California, Los Angeles (UCLA), Los Angeles, USA; 3 Pathology, David Geffen School of Medicine at University of California, Los Angeles (UCLA), Los Angeles, USA

**Keywords:** de quervain’s thyroiditis, subacute thyroiditis, thyroid fine needle aspiration, thyroid nodule, thyroid ultrasound

## Abstract

De Quervain’s thyroiditis (subacute thyroiditis) is a self-limited inflammation of the thyroid gland. It is diagnosed based on clinical history, physical examination, labs, and thyroid imaging. Thyroid fine needle aspiration (FNA) is typically not recommended unless the diagnosis is unclear or there are clinical signs concerning for malignancy. We present the case of a 75-year-old male who presented with symptoms of De Quervain’s thyroiditis and an inflammatory thyroid nodule. He had a thyroid FNA for further evaluation of the thyroid nodule. Ultimately, his clinical course was prolonged after he had a thyroid FNA, which was presumed to have contributed to further thyroid inflammation. This case highlights the importance of distinguishing De Quervain’s thyroiditis from other causes of thyroid pain, as well as the use of serial thyroid ultrasounds, as it may help avoid unnecessary FNA.

## Introduction

De Quervain’s thyroiditis (DQT), also known as subacute thyroiditis (SAT), is an inflammatory disorder of the thyroid gland that is self-limited and thought to be triggered by viral upper respiratory tract infections (URIs) [1]. The reported incidence is 12.1 cases per 100,000/year [2]. It typically presents with acute, unilateral thyroid pain that may radiate to the ears as well as the jaw. Systemic symptoms such as fatigue, fever, and pharyngitis are frequently reported in the prodromal phase. Hyperthyroidism symptoms may be present, and there is low radioactive iodine uptake while hyperthyroidism is present. Physical examination is typically notable for a firm, tender, and mildly to moderately enlarged thyroid [1].

De Quervain’s thyroiditis can present diagnostic challenges due to its overlap with other thyroid pathologies, including potentially life-threatening conditions such as suppurative thyroiditis or thyroid malignancies. These may share similar clinical features, including visible thyroid enlargement and localized pain. While thyroid fine needle aspiration (FNA) is generally not indicated for DQT, it is sometimes performed if the diagnosis is unclear [1].

Cytological findings in DQT typically reveal multinucleated giant cells, epithelioid histiocytes, inflammatory infiltrates, and disrupted follicular cells within a granulomatous background [3]. Although FNA can aid in differentiating DQT from other thyroid conditions, such as malignancies, it carries the risk of exacerbating the inflammatory process. Some complications of thyroid FNA that have been reported include local hematoma formation, pain, swelling, infection, recurrent laryngeal nerve or tracheal injury, and thyrotoxicosis [4].

This case study examines a 75-year-old male patient with DQT in whom FNA was performed due to concerns for malignancy. The biopsy was thought to have resulted in a marked exacerbation of his thyroid pain and swelling as well as prolonging his recovery. This case illustrates an important complication of thyroid FNA, which, to our knowledge, has not been previously reported in the literature. 

This case was presented as a poster in May 2025 by author Brendan Pelikh for the University of California, Los Angeles (UCLA) Undergraduate Research Week.

## Case presentation

**Figure 1:**
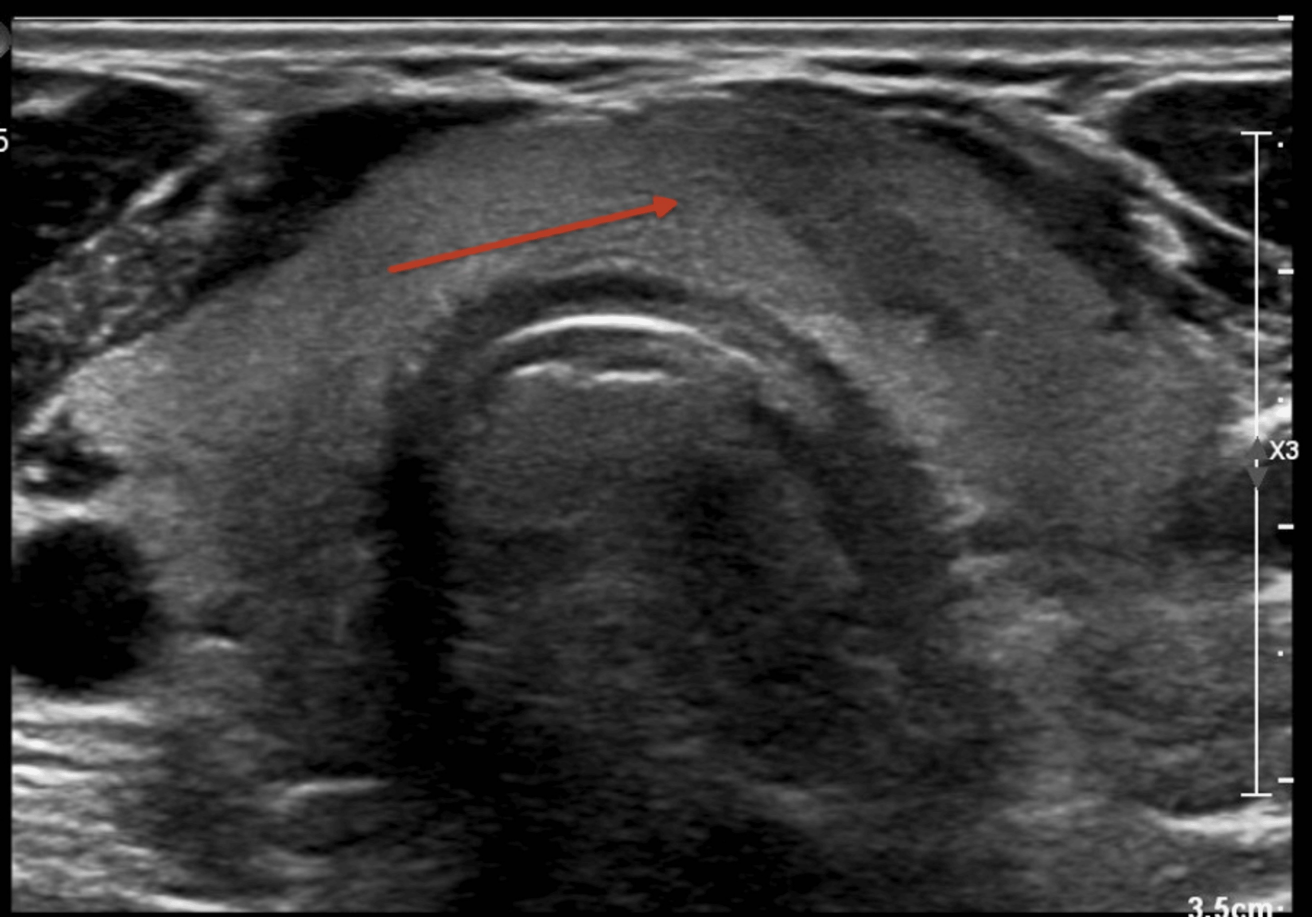
Initial thyroid ultrasound Demonstrates a 16 mm solid isthmus hypoechoic lesion (red arrow) thought to be a nodule versus focal heterogeneity.

**Figure 2:**
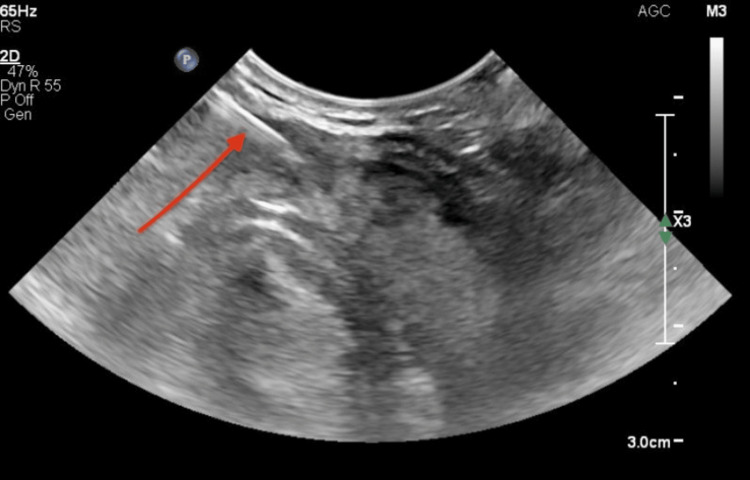
Thyroid ultrasound performed during FNA The needle used for the FNA appears as the white line (identified by the red arrow) entering the right of the thyroid nodule, which is located in the isthmus. FNA: Fine needle aspiration

**Figure 3:**
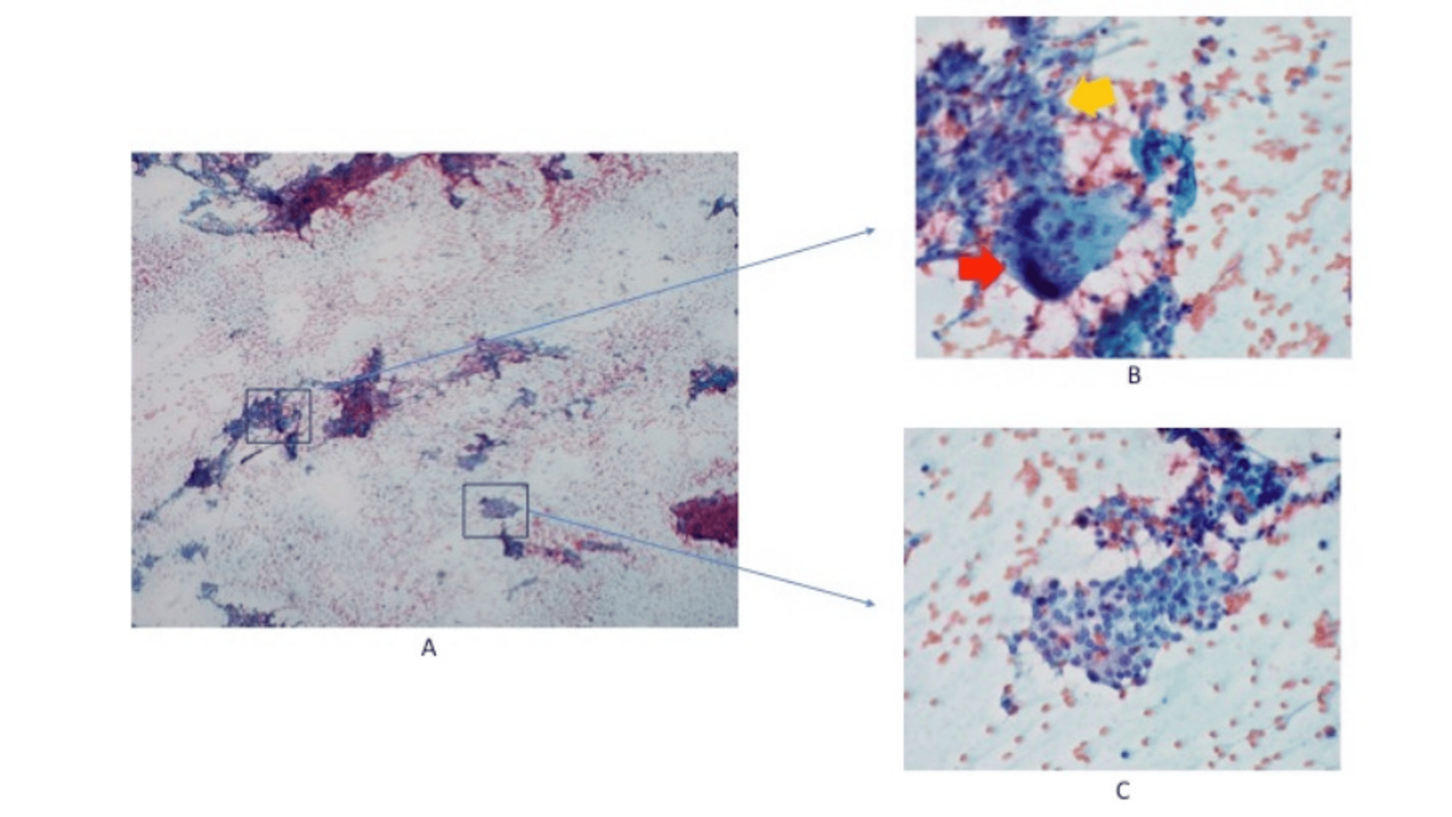
Cytology from the thyroid FNA A: A low-power view shows a moderately cellular specimen with several sheets of benign reactive follicular cells, histiocytes, mixed inflammatory cells, as well as some multinucleated giant cells (4x). B: Histiocytes, mixed inflammatory cells, and multinucleated giant cells (40x) (red arrow: multinucleated giant cells, yellow arrow: histiocytes); C: Reactive follicular cells (40x) FNA: Fine needle aspiration

A 75-year-old male with no history of thyroid dysfunction presented to the ear, nose, and throat (ENT) specialist with a three-week history of anterior neck pain localized to the thyroid region. He reported initially experiencing a URI approximately two weeks before the onset of anterior neck pain, palpitations, and fatigue. He had no difficulty breathing, hoarseness, fever, night sweats, or weight loss. On examination, the ENT specialist noted thyroid gland enlargement. There were no labs collected, and no radioactive iodine uptake scan was completed at this time. Thyroid ultrasound (Figure 1) demonstrated a 16 mm solid isthmus hypoechoic lesion, which the radiologist interpreted to be a nodule versus focal heterogeneity. The ENT ordered a thyroid FNA to rule out malignancy. The radiologist completed thyroid FNA via a parallel (longitudinal) approach (Figure 2). Cytology (Figure 3) demonstrated a moderately cellular specimen with several sheets of benign reactive follicular cells, histiocytes, and mixed inflammatory cells, as well as some multinucleated giant cells, consistent with DQT.

Initial treatment by the ENT specialist included a prednisone taper (initial dose of 60 mg daily was tapered over 16 days) and a course of azithromycin for a presumed acute bacterial URI. The pain initially subsided. The patient subsequently had a thyroid FNA. Following the thyroid FNA, the patient experienced a flare in symptoms, with increased neck pain and swelling. He was referred to endocrinology for further management. 

**Figure 4:**
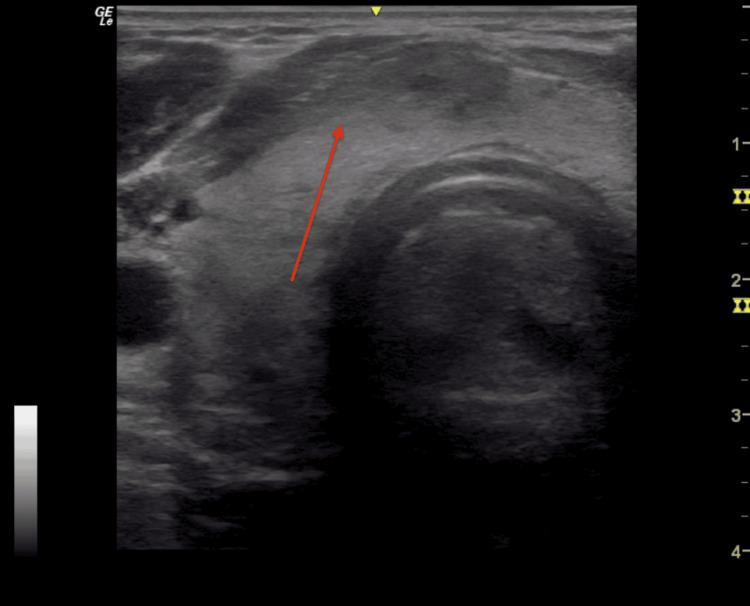
Thyroid ultrasound at first endocrinology appointment (one month after the FNA) Demonstrated a 16 mm hypoechoic region (red arrow) in the right lower isthmus, without calcifications, vascularity, or capsular invasion, which was consistent with trauma from the needle. There was no nodule at that time. The hypoechoic region noted on the initial ultrasound had resolved.

**Figure 5:**
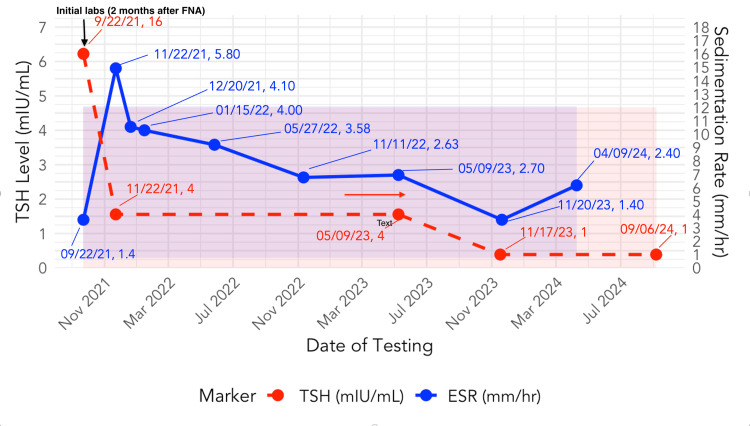
The trend of TSH and ESR levels over time The reference range for TSH is 0.3-4.7 mcIU/mL, and for ESR is ≤ 12 mm/hr (≤ 20 mm/h on Westergren assay). No labs are available from the initial presentation to the ENT specialist. However, there is an elevation of the TSH four months after the FNA with subsequent normalization of the TSH. There is an elevated ESR two months after the FNA, with subsequent normalization of the ESR. FNA: Fine needle aspiration, TSH: Thyroid-stimulating hormone, ESR: Erythrocyte sedimentation rate

At the initial endocrinology appointment, one month post-FNA, a thyroid ultrasound demonstrated a 16 mm hypoechoic region in the right lower isthmus, without calcifications, vascularity, or capsular invasion, which was consistent with trauma from the needle (Figure 4). There was no nodule at that time. The hypoechoic region noted on the initial ultrasound had resolved. The patient reported persistent pain described as coming in waves and tenderness to palpation over the thyroid. A second prednisone taper (initial dose of 40 mg daily) was prescribed, but his pain was only partially resolved after the two-week taper. Labs were ordered at that time and ultimately completed two months after the FNA, showing an elevated erythrocyte sedimentation rate (ESR) at 16 mm/hr (reference range is ≤12 mm/hr) but normal thyroid stimulating hormone (TSH), free thyroxine index (FT4 index), and free triiodothyronine index (FT3 index) (Figure 5). 

**Figure 6:**
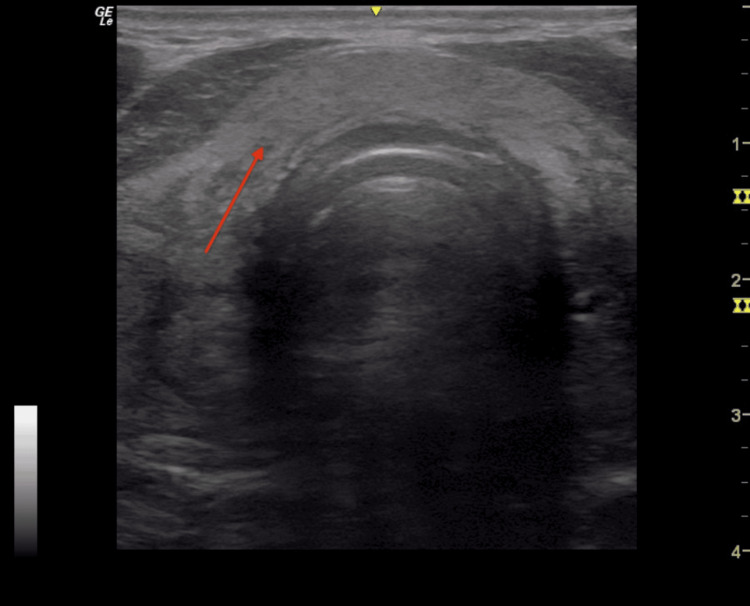
Thyroid ultrasound four months after the FNA No thyroid nodule present; the region of thyroid inflammation from the thyroid biopsy needle is resolving (arrow). The hypoechoic region noted on the initial ultrasound is not present. FNA: Fine needle aspiration

**Figure 7:**
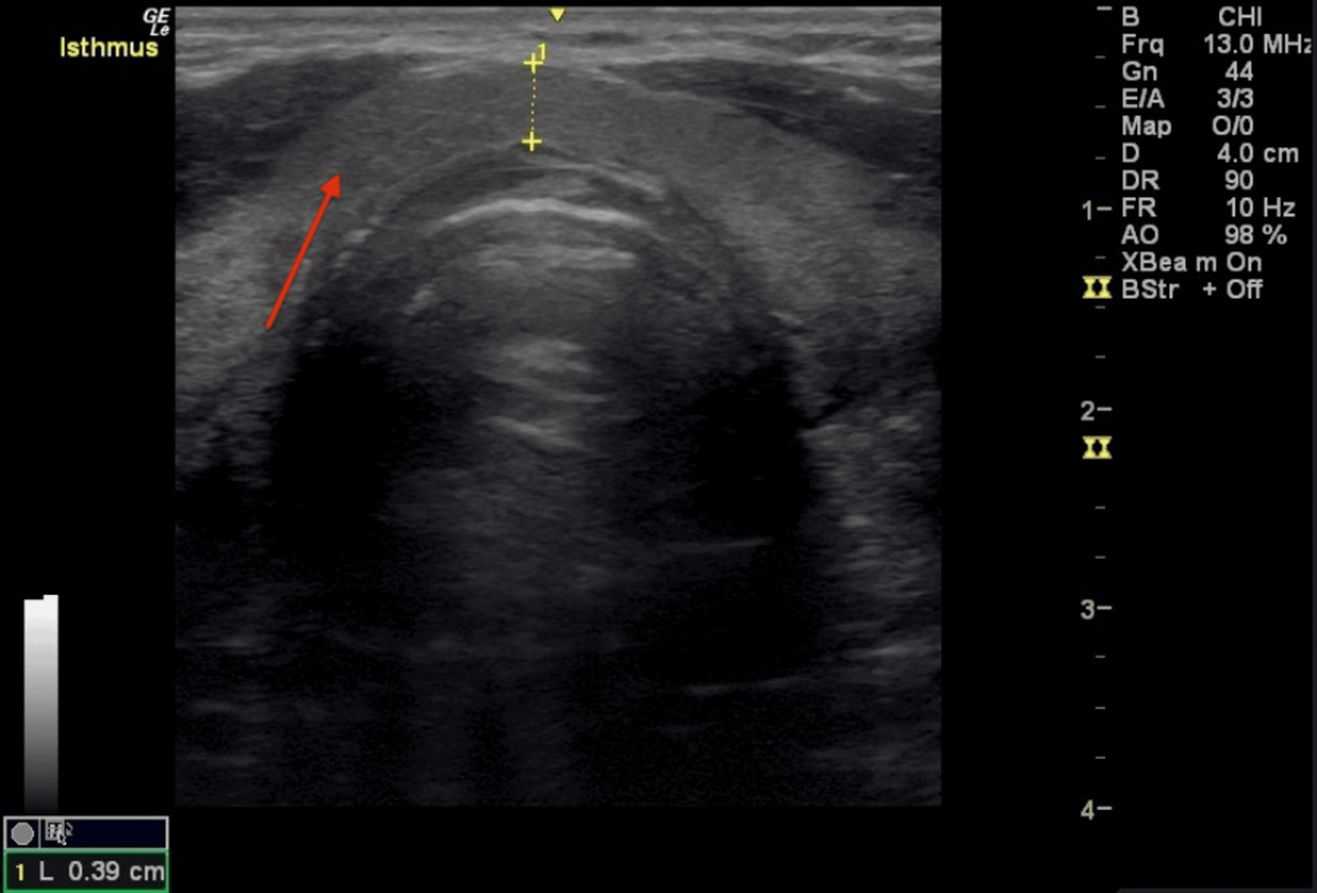
Thyroid ultrasound 11 months after the FNA The area of prior thyroid inflammation from the thyroid biopsy needle has resolved (arrow) and there are no thyroid nodules. FNA: Fine needle aspiration

Three ultrasounds were performed over the next 12 months, which showed a gradual reduction and eventual resolution of the thyroid inflammation from the biopsy needle, as well as no signs of a thyroid nodule (Figures 6-7). Ongoing lab monitoring revealed a transient hypothyroid phase four months post-FNA with TSH 5.8 mIU/L (reference range 0.3-4.7), normal FT4, normal FT3, and normal ESR (Figure 5), and the patient was asymptomatic at the time. This was followed by normalization of thyroid function testing (Figure 5). Table 1 summarizes the thyroid function test results and ultrasounds over time. Over the subsequent three years, the patient experienced intermittent neck discomfort and tenderness in the thyroid region that would spontaneously resolve within days or weeks. The patient continues to be regularly monitored. 

**Table 1:** Summary of the Thyroid function test results and thyroid ultrasounds over time TSH: Thyroid-stimulating hormone, FT4: Free thyroxine, FT3: Free triiodothyronine, ESR: Erythrocyte sedimentation rate

Laboratory Study	Initial presentation	One month post-FNA	Two months post-FNA	Four months post-FNA	Five months post-FNA	11 months post-FNA	Reference range
TSH			1.4 mcIU/mL	5.8 mcIU/mL	4.1 mcIU/mL		0.3-4.7
FT4			-	1.00 ng/dL	1.00 ng/dL		0.8-1.7
FT4 index			6.7	-	-		4.5-10.5
FT3			-	282 pg/dL	318 pg/dL		222-383
FT3 index			91.8	-	-		78.0-162.0
ESR			16 mm/hr	4 mm/hr	2 mm/hr*		≤ 12 mm/hr
							(*≤ 20 mm/h on Westergren assay)
Thyroid ultrasound	16 mm solid hypoechoic nodule versus heterogeneity in the isthmus	Hypoechoic region in the right lower isthmus consistent with needle trauma; no thyroid nodule		No thyroid nodule; post-biopsy inflammation is resolving		No thyroid nodule; post-biopsy inflammation has resolved	

## Discussion

We present a unique case of prolonged and recurrent DQT presumed to be from thyroid FNA, a finding that has not previously been reported in the literature. Our case demonstrates the importance of correctly identifying the diagnosis of DQT based on its clinical presentation so that it can be appropriately managed. Patients with DQT often initially present with URI symptoms, followed by anterior neck pain, thyroid enlargement, and tenderness [1,2]. Labs initially demonstrate hyperthyroidism (typically with a ratio of T3 to T4 that is less than 20) lasting up to six weeks, followed by transient hypothyroidism seen in about 30% of individuals, and then most individuals ultimately become euthyroid [1,5]. Additional lab findings include elevated inflammatory markers (ESR, C-reactive protein), mild anemia, leukocytosis, and potentially low titers of thyroid autoantibodies [1]. In our patient, we presume the labs would have shown hyperthyroidism initially, but unfortunately labs were not drawn at the initial ENT appointment. Labs collected at two months post-FNA demonstrated an elevated ESR, and subclinical hypothyroidism was present at four months post-FNA, which ultimately resolved. Radioactive iodine uptake and scans typically demonstrate low uptake during the hyperthyroid phase. On ultrasound, the thyroid appears heterogenous with hypoechoic areas, and Doppler demonstrates reduced flow while in the hyperthyroid state, as seen in our patient. De Quervain’s thyroiditis should be distinguished from acute infectious (suppurative) thyroiditis, which presents as more severe neck pain (which can be localized) with fever and leukocytosis [2]. A minority of patients have hyperthyroidism or hypothyroidism [2]. Thyroid imaging (ultrasound or CT) is typically diagnostic, demonstrating hypoechoic regions, effacement of the border between the thyroid and surrounding tissue, as well as abscess formation [1,2]. These findings were not present in our patient.

Uncommonly, Graves' disease can present with thyroid pain due to capsule stretch, but typically has detectable thyroid autoantibodies, increased uptake on radioactive iodine uptake and scan, and thyroid ultrasound demonstrates hypervascularity [6]. Thyroid lymphoma or other malignancy is also a consideration, as it can present with neck pain and an elevated ESR [7]. Thyroid lymphoma has characteristic findings, including a quickly enlarging thyroid, obstructive symptoms (from compression of the trachea, esophagus, neck veins, and recurrent laryngeal nerve), systemic symptoms (fever, night sweats, and weight loss), signs of thyroid dysfunction, and a firm, immobile thyroid goiter with lymphadenopathy [8]. Thyroid ultrasound demonstrates multiple hypoechoic lesions, hypervascularity, and posterior echoes. If ultrasound findings are inconclusive or suggestive of lymphoma, then biopsy may be indicated [7,8]. Our patient did not have features suggestive of malignancy. 

Our case highlights the role of serial thyroid ultrasound in cases of DQT, as this may help avoid unnecessary thyroid FNA. First, it is important to note that the American Thyroid Association (ATA) guidelines generally do not recommend FNA for evaluation of subacute thyroiditis, unless the diagnosis is unclear [1]. In our case, thyroid ultrasound also demonstrated a thyroid nodule described by radiology as thyroid imaging reporting and data system (TIRADS 4) because it was solid, hypoechoic, and had irregular margins but had no vascularity or calcifications. This would have been considered a high suspicion pattern based on the ATA criteria [9]. The initial thyroid ultrasound findings led to FNA, and serial ultrasounds showed resolution of the nodule. As the radiologist’s impression of the nodule is solely based upon the available images, it is important for clinicians to consider the clinical context when determining the necessity of FNA. Rather than immediately performing a thyroid FNA, the next step in this case should have been to obtain a TSH level [1,9]. It is also important for clinicians to understand the clinical course of thyroid nodules in patients with DQT. Observational studies have demonstrated various outcomes of thyroid nodules in individuals with DQT, ranging from some nodules resolving to instances of thyroid cancer being discovered [10,11]. Clinicians should consider waiting until the thyroiditis has improved to determine if ultrasound findings warrant doing a thyroid FNA. 

Our case also demonstrates that while FNA is generally safe, there are potential risks. Thyroid FNA is generally considered a reliable, accessible, and low-cost diagnostic tool [4]. While complications following FNA are infrequently reported and are rarely associated with significant morbidity, it is important to be aware of the known complications, including procedural discomfort, hemorrhage/hematomas, thyroid swelling, thyroid infection, recurrent laryngeal nerve palsy, and vasovagal reactions [4]. Observational studies evaluating pain persisting following the procedure report persistent symptoms in less than 1% of patients [4]. However, the true incidence of more serious complications may be underrepresented in the literature due to potential reporting bias or concern over medico-legal complications [4].

Our case is unique in that the patient’s FNA was thought to have contributed to a complication of prolonged thyroid inflammation. To our knowledge, this has not been previously reported in the literature. De Quervain’s thyroiditis is a self-limited condition in which the inflammatory thyrotoxic phase typically resolves by six weeks. Our patient continued to demonstrate signs of thyroid inflammation months after the onset of symptoms. While the recurrence rate of subacute thyroiditis is generally estimated to be low in the literature (1% to 4%), our patient has also continued to experience flares over the subsequent three years [1]. This patient’s prolonged inflammation and recurrences were presumed to be due to irritation and inflammation caused by the FNA. It is therefore important to weigh the risks and benefits of FNA and individualize the decision for FNA on a case-by-case basis in nodules that meet FNA criteria. 

## Conclusions

This case highlights an uncommon prolonged course of DQT, with inflammation likely exacerbated by thyroid FNA. Important clinical features to consider when diagnosing DQT include the patient's symptoms, physical examination, serial ultrasound imaging, and thyroid function tests. Before performing an invasive procedure that could exacerbate DQT, clinicians must complete a thorough clinical assessment. 
